# Is acidemia at birth a risk factor for functional gastrointestinal disorders?

**DOI:** 10.1007/s00431-022-04565-x

**Published:** 2022-08-04

**Authors:** Flavia Indrio, Flavia Marchese, Matteo Rinaldi, Gianfranco Maffei, Vanessa Dargenio, Roberta Cinquepalmi, Massimo Pettoello Mantovani, Arianna Aceti

**Affiliations:** 1 Department of Medical and Surgical Science, Pediatric Section, University of Foggia, Viale Pinto 1, 71122 Foggia, Italy; 2 Department of Neonatology and NICU, Ospedali Riuniti Foggia, Viale Pinto 1, 71122 Foggia, Italy; 3 European Pediatric Association, Union of National European Pediatric Societies and Associations, Berlin, Germany; 4 Association Pour L’Activité Et La Recherche Scìentifiques, Nouchatel, Switzerland; 5 University of Foggia, Foggia, Italy; 6 Italian Society of Pediatrics, Rome, Italy; 7 Italian Academy of Pediatrics, Milan, Italy; 8 Department of Medical and Surgical Sciences, University of Bologna, Bologna, Italy; 9 Neonatal Intensive Care Unit, IRCCS AOUBO, Via Massarenti 9, 40138 Bologna, Italy

**Keywords:** Functional gastrointestinal disorders, Acidemia, Cord blood pH, Newborn

## Abstract

Functional gastrointestinal disorders (FGIDs) are common in early childhood. It has been demonstrated that neonatal acidemia at delivery can lead to significant neonatal morbidity. The primary aim of this study was to evaluate the relationship between acidemia at birth and the development of FGIDs, as regurgitation, colic, and constipation, in term infants. Term newborns born at the Foggia University Hospital, Italy during the year 2020 were included in the study. As per routine clinical practice, a cord blood gas analysis on a blood sample drawn from the umbilical artery (UA) of each infant immediately after birth was performed, and Apgar score was recorded. One year after birth, each infant’s parents were interviewed through a phone call to investigate development of FGIDs, feeding practices, and morbidities. During the study period, 1574 term newborns met the inclusion criteria. The prevalence of infantile colic, regurgitation, and constipation was higher in infants with low UA pH (colic 51.5% vs. 25.4%, *p* < 0.001; regurgitation 30.6% vs. 15.2%, *p* < 0.001; constipation 24.6% vs. 16.0%, *p* = 0.015), with infants having moderate-severe acidemia facing the highest risk for all the examined FGIDs. In binary logistic regression analyses, UA pH and perinatal antibiotic exposure proved to be independently associated with the later diagnosis of each FGID.

*Conclusion*: Newborns with acidemia at birth appear to face a higher risk of FGIDs in infancy. Avoiding low cord blood pH should continue to be the goal for obstetricians, while enhanced long-term surveillance for infants who experienced birth acidemia should be required.

**Table:** 

**What is Known:** *• Cord blood gas analysis is recommended in all high-risk deliveries, and in some centers, it is performed after all deliveries.* *• Neonatal acidemia at birth has been linked to adverse outcomes, mainly neurological. Recently, perinatal asphyxia has been reported to increase the risk of developing necrotizing enterocolitis in term infants.*	
**What is New:** *• An association between acidemia at birth and risk of developing FGIDs such as regurgitation and colic during the first year of life had never been described so far.* *• An increased surveillance of infants with low UA pH at birth may be beneficial and could allow for early detection of any of the reported FGIDs.*

**What is Known:**

*• Cord blood gas analysis is recommended in all high-risk deliveries, and in some centers, it is performed after all deliveries.*

*• Neonatal acidemia at birth has been linked to adverse outcomes, mainly neurological. Recently, perinatal asphyxia has been reported to increase the risk of developing necrotizing enterocolitis in term infants.*

**What is New:**

*• An association between acidemia at birth and risk of developing FGIDs such as regurgitation and colic during the first year of life had never been described so far.*

*• An increased surveillance of infants with low UA pH at birth may be beneficial and could allow for early detection of any of the reported FGIDs.*

## Introduction

Functional gastrointestinal disorders (FGIDs) are defined as a variable combination of age-dependent, chronic, or recurrent symptoms not explained by structural or biochemical abnormalities [1]. They represent a common disorder in early childhood [2].

The pathogenesis of these diseases has yet to be fully elucidated, but it is plausible that several factors in early life could play a role in predisposing to FGIDs.

It has been suggested that acidemia at birth, defined as an umbilical artery (UA) pH below normal ranges [3–5], might be associated with clinically relevant neonatal outcomes, such as mortality and neurological diseases, including hypoxic-ischemic encephalopathy, intraventricular hemorrhage, and cerebral palsy [3]. Cord blood gas analysis is an accurate and validated tool for objective assessment of the metabolic status of the newborn at the time of delivery; however, cord blood analysis is not universally performed, so it is possible to miss a neonatal acidemia when the clinical condition and Apgar score are in the normal range [6]. UA blood sampling is preferable to umbilical vein blood sampling because arterial pH and baseline deficit (BD) provide the most accurate information on fetal acid–base status and correlate better with newborn morbidity [7].

Currently, there are no clear threshold values for normal cord blood pH, BD, and lactate; however, it is well recognized that the occurrence of neonatal morbidity is inversely related to UA pH, with the highest risk at the lowest pH [3]. In most studies, a UA pH below 7.0 and a BE ≥ 12 mmol/L are described to be strongly associated with neonatal morbidities [3, 4], such as respiratory distress and admission to neonatal intensive care units [6]. In addition, it has recently been shown that UA pHs below 7.20 are also associated with an increased risk of composite neonatal morbidity, including respiratory distress and sepsis [5].

To date, an association between acidemia at birth and gastrointestinal (GI) disorders has not been reported. However, a potential relationship between low UA pH and feeding problems is likely, perhaps involving alterations in intestinal blood flow soon after birth [8]. Nor has an association with low cord blood pH and the development of FGIDs been reported to date.

The purpose of the present study was to evaluate whether low UA pH was related to the development of FGIDs in infancy.

## Materials and methods

### Participants and instruments

This retrospective, observational cohort study was conducted at the Ospedale Riuniti in Foggia, Italy. Data were collected from all deliveries occurring at term from January 1 to December 31, 2020. The study was approved by the Ethics and Research Committee of the Azienda Ospedaliera Universitaria, and mothers provided written informed consent before data collection and cord blood collection. The study was conducted in accordance with the principles of the Declaration of Helsinki.

Inclusion criteria were single birth, gestational age (GA) ≥ 37 weeks, and birth weight adjusted for GA. Infants born preterm, small or large for GA, or with any major congenital anomaly were excluded from the analysis, as were infants with clinical conditions requiring admission to the neonatal unit. In addition, data from infants born to mothers with pre-eclampsia, hypertension, gestational or pregestational diabetes, or thyroid disease were excluded.

In the delivery room, the attending physician or pediatric nurse gave the Apgar score at 1 and 5 min after birth. A UA blood sample was collected for each delivery, as per routine clinical practice in our hospital. Cord blood samples were obtained by cutting a section of the umbilical cord twice immediately after each delivery. Arterial blood was drawn in a 2-mL preheparinized syringe under aseptic conditions and analyzed by a blood gas analyzer (GEM Premier 4000, Instrumentation Laboratory, Milan, Italy) within 15 min after delivery. In order to include both mild and severe cases, the diagnosis of acidemia was made when the UA pH was less than 7.20 [5].

Data on delivery, anthropometric measurements of infants at birth, and clinical conditions were collected from medical records.

One year after birth, parents of each infant were interviewed by telephone to investigate whether the infants had been diagnosed with FGID, feeding practices, and medical conditions. Specifically, parents were asked whether their infants had developed symptoms during the first year of life of any of the following FGIDs: infantile colic, regurgitation, and constipation, and whether these symptoms had been evaluated clinically by a pediatrician or pediatric gastroenterologist. In addition, parents were required to provide information on feeding at discharge from the nursery, occurrence of neonatal complications and treatment, administration of antibiotics and/or probiotics in the neonatal period, length of hospitalization, family history of allergy, and FGIDs.

### Statistical analysis

Statistical analyses were performed using IBM SPSS Statistics for Windows, version 27.0 (IBM Corp., Armonk, NY, USA). Data distribution was evaluated using the Kolmogorov–Smirnov test. As most variables followed a non-normal distribution, non-parametric tests were used. Categorical variables were expressed as number (percentage) and continuous variables as median (range).

First, we analyzed which variables differed between infants with UA pH ≤ 7.20 and > 7.20 in a univariate analysis using Fisher’s exact test for categorical data, with pairwise *z*-tests and Bonferroni correction where appropriate, and Mann–Whitney test for continuous data. Variables which proved to be significantly different between groups were used to build a binary logistical regression model using UA pH as dependent variable.

The rate of each FGID according to UA pH subgroup was estimated using Fisher’s exact test. In addition, potential confounders in the relationship between UA pH and each FGID were evaluated using Fisher’s exact test for categorical data, with pairwise *z*-tests and Bonferroni correction where appropriate, and Mann–Whitney test for continuous data. Variables which proved to be significantly different between groups were used to build different binary logistical regression models using each FGID as dependent variable. Potential correlation between the independent variables included in the binary logistic regression models was checked using the Spearman correlation test. A *p* value < 0.05 was considered statistically significant.

## Results

### Study population

**Fig. 1:**
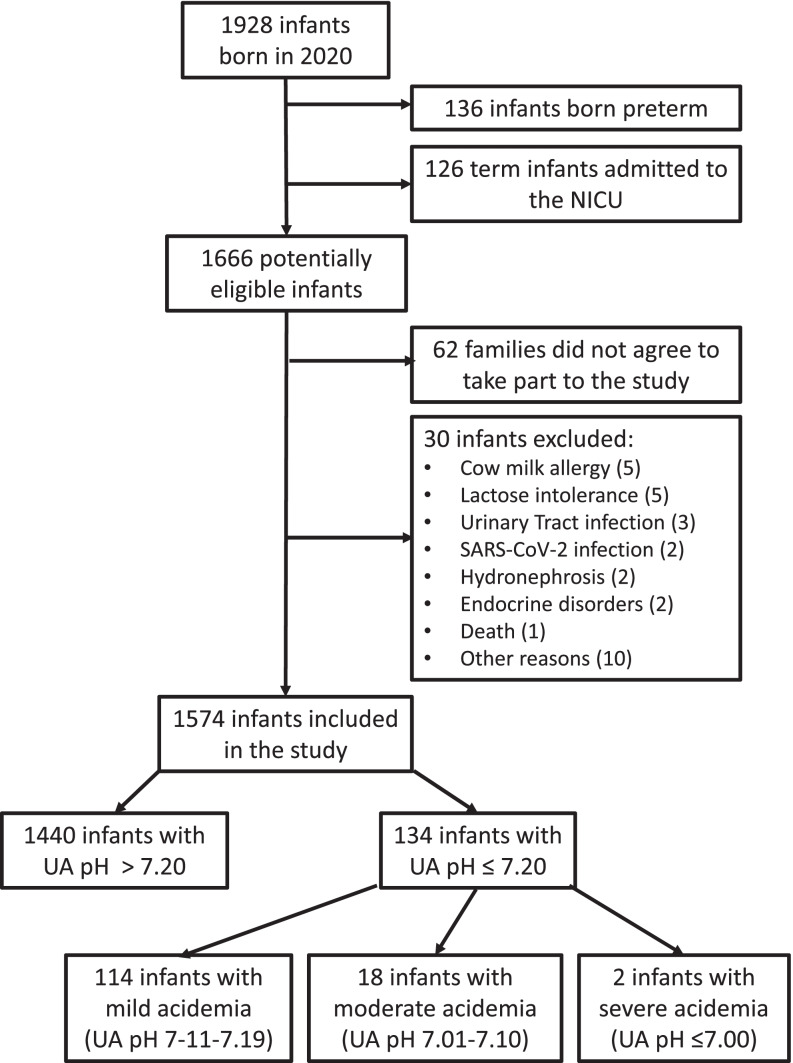
Flowchart of the study population

During the study period, there were 1928 deliveries in our hospital, with 1574 full-term newborns meeting the inclusion criteria of the study (Fig. 1). Among them, 134 infants had an umbilical artery pH ≤ 7.20.

The clinical and demographic characteristics of the study population, overall and according to UA pH, are shown in Table 1.

**Table 1:** Demographic and clinical characteristics of the study population, overall and according to cord blood pH; a *p* value < 0.05 was considered statistically significant. Categorical variables are expressed as number (percentage), while continuous variable as median (range)

	Overall population	pH > 7.20	pH ≤ 7.20	*p* value
Number of infants	1574	1440	134	
Female gender	748 (47.5)	690 (47.9)	58 (43.3)	0.321
Vaginal delivery	941 (59.8)	851 (59.1)	90 (67.2)	0.029
Nulliparous	756 (48.0)	677 (47.0)	79 (59.0)	0.009
Birth weight, g	3300 (2200–4780)	3300 (2200–4780)	3285 (2500–4360)	0.404
Gestational age, weeks	39 (37–42)	39 (37–42)	39 (38–42)	< 0.001
Apgar score at 1′	8 (5–9)	8 (5–9)	8.00 (5–9)	< 0.001
Apgar score at 5′	9 (6–10)	9 (6–10)	9 (7–10)	< 0.001
Mode of feeding at discharge				0.084
Exclusive breastfeeding Partial breastfeeding Exclusive formula feeding	300 (19.1) 1178 (74.8) 96 (6.1)	75 (19.1) 1083 (75.2) 82 (5.7)	25 (18.7) 95 (70.9) 14 (10.4)	
Antibiotic exposure in the perinatal period	160 (10.1)	149 (10.3)	11 (8.2)	0.605

Proportion of nulliparous mothers and rate of vaginal delivery were significantly higher in infants with UA pH ≤ 7.20; in addition, these infants had slightly higher GA (mean 39.5 vs. 39.1 weeks, *p* < 0.001) and lower Apgar scores both at 1 and 5 min after birth (mean 7.8 vs. 8.1 and 8.7 vs. 8.9, respectively, *p* < 0.001 for both comparisons).

A logistic regression model was built including UA pH as dependent variable and parity, mode of birth, and GA as covariates. Apgar scores at 1 and 5 min were not included in the regression model as they were correlated both one with the other (correlation coefficient 0.575, *p* < 0.001), and also with UA pH (correlation coefficient 0.134 and 0.145, respectively; *p* < 0.001 for both comparisons). Both parity and GA proved to be independently correlated with UA pH (*p* < 0.05 for both—Table 2).

**Table 2:** Binary logistic regression models built up to evaluate factor potentially associated with umbilical artery (UA) pH (model 1) and potential confounders in the relationship between UA pH and each functional gastrointestinal disorders (colic—model 1, regurgitation—model 2, constipation—model 3). A *p* value < 0.05 was considered statistically significant

**Model 1 UA pH**					
Parameter	*B*	Standard error	Wald	Sig	Exp(B)
Delivery mode	.294	.209	1.981	.159	1.342
Parity	.444	.190	5.459	*.019*	1.560
Gestational age	.227	.085	7.068	*.008*	1.255
Constant	− 11.735	3.320	12.494	.000	.000
**Model 2 Colic**					
Parameter	*B*	Standard error	Wald	Sig	Exp(B)
Antibiotics	.726	.167	18.864	*.000*	2.066
Parity	.353	.116	9.273	*.002*	1.423
Cord blood pH	− 1.138	.185	37.728	*.000*	.320
Constant	− .205	.188	1.187	.276	.815
**Model 3 Regurgitation**					
Parameter	*B*	Standard error	Wald	Sig	Exp(B)
Antibiotics	.730	.186	15.373	*.000*	2.075
Parity	.366	.139	6.943	*.008*	1.441
Cord blood pH	− .891	.204	19.112	*.000*	.410
Constant	− 1.108	.210	27.957	.000	.330
**Model 4 Constipation**					
Parameter	*B*	Standard error	Wald	Sig	Exp(B)
Antibiotics	.699	.185	14.275	*.000*	2.011
Cord blood pH	− .930	.203	21.088	*.000*	.394
Constant	− .882	.189	21.748	.000	.414

### Functional gastrointestinal disorders

The overall prevalence of infant colic was 27.6% (434/1574 infants); the prevalence of regurgitation was 16.5% (260/1574), and constipation was 16.7% (263/1574); the prevalence of the examined FGIDs was similar to that reported in the literature [9, 10].

**Fig. 2:**
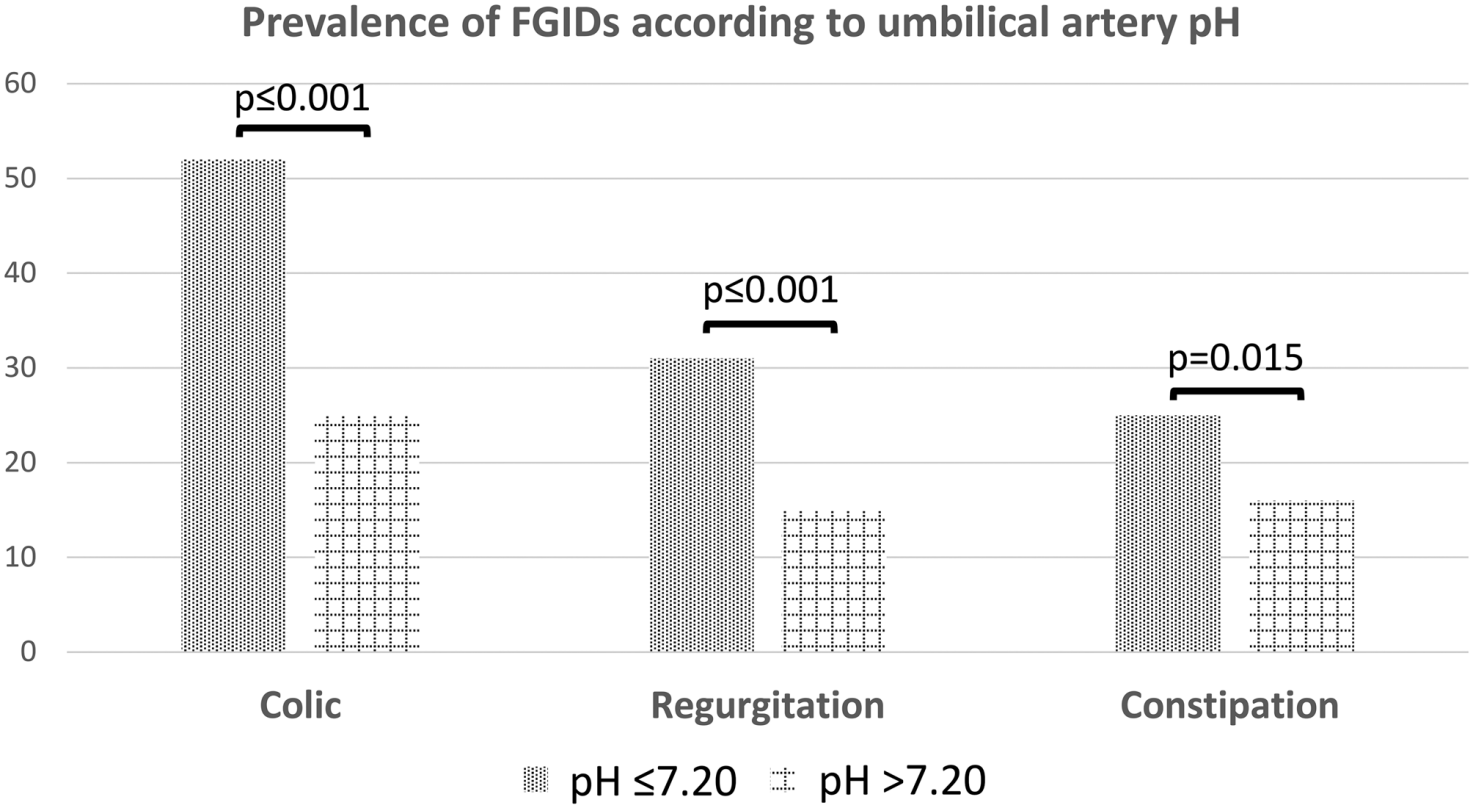
Prevalence of functional gastrointestinal disorders (FGIDs) in the study population according to umbilical artery (UA) pH. Prevalence is expressed as percentage

The prevalence of infantile colic, regurgitation, and constipation was different between infants with and without acidemia (colic 51.5% vs. 25.4%, *p* < 0.001; regurgitation 30.6% vs. 15.2%, *p* < 0.001; constipation 24.6% vs. 16.0%, *p* = 0.015), with infants having lower UA pH at greater risk of experiencing FGIDs (Fig. 2).

Infants with colic were more likely to have been exposed to antibiotics in the perinatal period (15.9% vs. 8.3%, *p* < 0.001) and to be born to nulliparous mothers (54.6% vs. 45.6%, *p* = 0.002). No differences in terms of gender, mode of delivery, GA, birth weight, Apgar score, and type of neonatal feeding were documented. In binary logistic regression analysis, UA pH, perinatal antibiotic exposure, and parity proved to be independently associated with the later diagnosis of colic (Table 2).

Infants with regurgitation were more likely to have been exposed to antibiotics in the perinatal period (15.8% vs. 9.1%, *p* < 0.001), to be born to nulliparous mothers (55.8% vs. 46.5%, *p* = 0.007). No differences in terms of gender, mode of delivery, GA, birth weight, Apgar score, and type of neonatal feeding were documented. In binary logistic regression analysis, UA pH, perinatal antibiotic exposure, and parity proved to be independently associated with the later diagnosis of regurgitation (Table 2).

Infants with constipation were more likely to have been exposed to antibiotics in the perinatal period (16.1% vs. 9.4%, *p* < 0.001). No differences in terms of gender, mode of delivery, parity, GA, birth weight, Apgar score, and type of neonatal feeding were documented. In binary logistic regression analysis, both UA pH and perinatal antibiotic exposure proved to be independently associated with the later diagnosis of constipation (Table 2).

In order to evaluate whether a sort of dose–response relationship existed between UA pH and each FGID, an additional subgroup analysis was performed by stratifying infants into three different groups, defined as normal pH (pH > 7.20), mild acidemia (pH 7.11–7.19, 114 infants), and moderate-severe acidemia (pH ≤ 7.10, 20 infants), as the further distinction between moderate and severe acidemia did not seem feasible (only 2 infants in the severe group). This subgroup analysis demonstrated that the rates of colic (25.4%, 43.9%, 55.0%, respectively) and regurgitation (15.2%, 29.8%, 35.0%, respectively) were significantly different across subgroups (*p* < 0.001 for both), with infants having moderate-severe acidemia facing the highest risk of both colic and regurgitation. As for constipation, significant differences were documented between the normal pH and the moderate-severe acidemia subgroups (13.9 vs. 40.0%, *p* = 0.005), but not between any of them and the mild acidemia subgroup (21.9%).

## Discussion

The results of the present study showed an association between acidemia at birth and risk of developing FGIDs, such as colic, regurgitation, and constipation, during the first year of life, with infants having moderate-severe acidemia facing the highest risk. The prevalence of these diseases in our population was similar to that reported in the literature [9, 10]. The association between acidemia at birth and FGIDs proved to remain statistically significant also after correcting for potential confounders; in addition, the occurrence of the examined FGIDs proved to be independently associated with perinatal antibiotic exposure for all the three examined FGIDs, and also with nulliparity for colic and regurgitation.

These findings are in line with recent literature: In a systematic review [11], the potential role of neonatal antibiotic exposure in predisposing to FGIDs and also to chronic GI diseases, such as coeliac disease and inflammatory bowel disease in the first 2 years of life, has been described. In addition, the same authors have suggested a potential link between antibiotic exposure and some FGIDs, such as functional abdominal pain, later on, at 4–6 years of age [12]. The role of parity as a potential risk factor for later FGIDs has also been described: In a population-based study performed in Colombia, being an only child or the first-born in the family was associated with a higher risk of developing some FGID, such as constipation, and this finding was hypothesized by the authors to relate to an increased attention to potential symptoms of FGIDs in parents of an only child [13].

Cord blood gas analysis is recommended in all high-risk deliveries and in some centers, it is performed after all deliveries [14]. In neonatal units where cord blood gas analysis is not routinely performed, clinical conditions immediately after birth are assessed primarily by Apgar score. However, it has been suggested that the correlation between Apgar score and cord blood pH is not always optimal. The Apgar score has some known limitations, which are subjectivity and dependence on several factors including maternal sedation or anesthesia, congenital malformations, gestational age, and trauma. The healthy preterm infant, even without evidence of asphyxia, may still receive a low score for immaturity [15, 16]; moreover, a low score alone cannot predict morbidity or mortality for any individual infant. It is important to recognize the limitations of the Apgar score to use it appropriately. Integration of the Apgar score with umbilical cord blood gas analysis has the potential to improve neonatal assessment at birth, especially for infants who have an Apgar score within normal ranges. In fact, it has been observed that infants with a reassuring Apgar score may still have a risk of neonatal acidemia [6].

Neonatal acidemia occurs in 1–2% of deliveries. The results of the systematic review and meta-analysis performed by Malin et al. on the outcomes of more than 480,000 newborns showed that low UA pH was strongly associated with neonatal mortality and neurological sequelae (hypoxic-ischemic encephalopathy, intraventricular hemorrhage or periventricular leukomalacia, and cerebral palsy). Furthermore, low pH UA has been shown to be no less [17] or even better [18] in predicting adverse neonatal outcomes than UA BD.

While the link between neonatal acidemia and neurological problems is established, little information is available regarding the impact that low pH UA might have on GI disease. The process of hypoxia–ischemia and reperfusion is characterized by energy depletion, accumulation of extracellular glutamate, and activation of glutamate receptors, leading to a deleterious cascade of events resulting in neuronal death [19]. In addition, perinatal asphyxia has been reported to increase the risk of developing necrotizing enterocolitis (NEC) in term infants [20].

Recently, the role of toll-like receptor 4 (TLR4) signaling, which recognizes lipopolysaccharide on gram-negative bacteria, has been highlighted not only in the pathogenesis of NEC, but also in intestinal inflammation and inflammatory bowel disease [21]. Hypoxia, infection, and prematurity accentuate TLR4 expression in the intestinal mucosa creating an imbalance between pro-inflammatory and anti-inflammatory signaling. TLR4 is subsequently activated by enteric bacteria, triggering an inflammatory cascade that results in increased intestinal mucosal damage and reduced epithelial repair, which in turn promotes translocation of bacteria into the lamina propria, exacerbating intestinal inflammation [22]. Notably, Lactobacillus reuteri DSM 17,938 has been shown to decrease TLR4 expression in the intestine of mice and, consequently, the secretion of pro-inflammatory cytokines such as TNF-α and IL-1β by acting on the NF-kB pathway [23]. Through this mechanism, a reduction in the incidence of NEC in mice and an improvement in survival and disease severity were determined.

Although the relationship between the gut microbiota and the immune system has been studied over the years, few data are available for FGIDs. However, interestingly, in the study by Savino et al. supplementation in infants of the same strain of probiotic (Lactobacillus reuteri DSM17938) induced anti-inflammatory actions by increasing mRNA levels of regulatory T cells, a component of the adaptive immune system, although it did not appear to affect TLR expression [24].

Understanding whether and how hypoxia and acidemia may affect the functions of specific TLRs is important to find useful compounds to restore intestinal homeostasis.

The relationship between alterations in intestinal perfusion and oxygenation after fetal hypoxia and the development of feeding intolerance and NEC has been examined in several studies. It is known that the fetus responds to hypoxia by redistributing blood flow to essential vascular beds, a process known as brain sparing [25].

Reduced blood flow to the stomach, small intestine, and large intestine after hypoxia has been demonstrated in newborn piglets and lambs [26]. In preterm infants, an association has been documented between low splanchnic oxygenation and the development of feeding intolerance and NEC [27], and a low ration of splanchnic and brain oxygenation after feeding in infants who later develop feeding intolerance [28]. In addition, Chen et al. observed a correlation between superior mesenteric artery (SMA) blood flow and UA blood gases, with decreased SMA blood flow in the presence of UA acidemia [29], and Kempley et al. observed a relationship between SMA blood flow characteristics and NEC progression [30]. It is plausible that low splanchnic blood flow and subsequent splanchnic hypoxia may play a role in the development of early and possibly late GI complications, including some FGIDs.

Matara and co-workers, in a recent systematic review [31], suggested that the GI tract is a target organ, with high sensitivity to ischemia–hypoxia, and even short periods of ischemia may cause significant local tissue damage. Fetal hypoxia and perinatal asphyxia reduce bowel motility, especially in preterm neonates. This could also be another possible explanation of the early damage to the acidemic newborn and the development of FGIDs in the acidemic newborn.

The present study has some limitations. First, compared with previously published studies examining the relationship between cord blood gas parameters and neonatal outcomes, our sample size was relatively small. Furthermore, given the multifactorial etiology of FGIDs, it is likely that additional factors, other than those examined in the present study, may contribute to disease risk.

The strength of the present study is based on the institutional policy of universal cord blood gas analysis in all newborns, which allowed us to examine the relationship between low UA pH and the occurrence of FGIDs in the entire sample.

Based on our research, increased surveillance of infants with low UA pH at birth may be beneficial and could allow for early detection of any of the reported FGIDs. Therefore, UA pH measurement detected at birth could play a predictive role for the development of FGIDs in childhood.

We are aware of the limitation of this study, which will not address the full question on the role of acidemia in the long-term development of FGIDs. However, the results of this pilot study may open new hypotheses to shed light on the complex multifactorial pathophysiology of FGIDs and try to identify, with large and well-designed prospective studies, other early risk factors that could be easily identified, and give the possibility to prevent the onset of FGIDs in childhood.

## Data Availability

Not applicable.

## Abbreviations

BD: Base deficit
CS: Cesarean section
FGIDs: Functional gastrointestinal disorders
GA: Gestational age
GI: Gastrointestinal
NEC: Necrotizing enterocolitis
SMA: Superior mesenteric artery
TLR4: Toll-like receptor 4
UA: Umbilical artery
VD: Vaginal delivery
